# Large scale genome-wide association and LDLA mapping study identifies QTLs for boar taint and related sex steroids

**DOI:** 10.1186/1471-2164-12-362

**Published:** 2011-07-13

**Authors:** Eli Grindflek, Sigbjørn Lien, Hanne Hamland, Marianne HS Hansen, Matthew Kent, Maren van Son, Theo HE Meuwissen

**Affiliations:** 1 NORSVIN (The Norwegian Pig Breeders Association), PO Box 504, 2304 Hamar, Norway; 2 Department of Animal and Aquacultural Sciences, Norwegian University of Life Sciences, PO Box 5003, 1432 Ås, Norway; 3 Centre for Integrative Genetics (CIGENE), Norwegian University of Life Sciences, PO Box 5003 Ås, Norway

## Abstract

**Background:**

Boar taint is observed in a high proportion of uncastrated male pigs and is characterized by an unpleasant odor/flavor in cooked meat, primarily caused by elevated levels of androstenone and skatole. Androstenone is a steroid produced in the testis in parallel with biosynthesis of other sex steroids like testosterone and estrogens. This represents a challenge when performing selection against androstenone in breeding programs, without simultaneously decreasing levels of other steroids. The aim of this study was to use high-density genome wide association (GWA) in combination with linkage disequilibrium-linkage analysis (LDLA) to identify quantitative trait loci (QTL) associated with boar taint compounds and related sex steroids in commercial Landrace (n = 1,251) and Duroc (n = 918) breeds.

**Results:**

Altogether, 14 genome wide significant (GWS) QTL regions for androstenone in subcutaneous fat were obtained from the LDLA study in Landrace and 14 GWS QTL regions in Duroc. LDLA analysis revealed that 7 of these QTL regions, located on SSC 1, 2, 3, 7 and 15, were obtained in both breeds. All 14 GWS androstenone QTLs in Landrace are also affecting the estrogens at chromosome wise significance (CWS) or GWS levels, while in Duroc, 3 of the 14 QTLs affect androstenone without affecting any of the estrogens. For skatole, 10 and 4 QTLs were GWS in the LDLA analysis for Landrace and Duroc respectively, with 4 of these detected in both breeds. The GWS QTLs for skatole obtained by LDLA are located at SSC 1, 5, 6, 7, 10, 11, 13 and 14.

**Conclusion:**

This is the first report applying the Porcine 60 K SNP array for simultaneous analysis of boar taint compounds and related sex hormones, using both GWA and LDLA approaches. Several QTLs are involved in regulation of androstenone and skatole, and most of the QTLs for androstenone are also affecting the levels of estrogens. Seven QTLs for androstenone were detected in one breed and confirmed in the other, i.e. in an independent sample, although the majority of QTLs are breed specific. Most QTLs for skatole do not negatively affect other sex hormones and should be easier to implement into the breeding scheme.

## Background

In spite of the increasing social pressure against it, surgical castration is still commonly performed to eliminate boar taint in male slaughter pigs. However, entire male pigs show better feed consumption, leaner meat percentage and better carcass traits than barrows and are more profitable for swine producers [e.g. 1]. An alternative to castration would therefore be highly advantageous for pig breeders worldwide. Boar taint is a characteristic and unpleasant odor and flavor noticeable when cooking meat from some uncastrated male pigs. The taint has been described as being akin to urine and manure [2] and is mainly caused by high levels of androstenone, skatole and/or indole in adipose tissue. The incidence of tainted carcasses varies considerably between breeds and the contribution of androstenone and skatole to boar taint has also been shown to be very different among breeds [e.g. 3]. Heritability of androstenone is high, ranging from 0.25 to 0.88 [3,4]. Androstenone is a natural steroid produced by the Leydig cells of the testis along with other sex steroids like estrogens and testosterone [5], but it is subsequently concentrated and converted in the salivary gland to an active sex pheromone. While estrogens are well known as important female sex hormones, they are also important controllers for the normal function of the adult male reproductive tract and male fertility (reviewed by Hess [6]) and serve as indicator of sexual maturity status in boars [7,8]. Therefore to identify genomic regions simultaneously affecting levels of boar taint and fertility related compounds, the sex hormones 17β-estradiol (one of the main estrogens), estrone sulphate (the reservoir for the active compound of 17β-estradiol) and testosterone (essential to maintain spermatogenesis and male fertility) [reviewed by 9] were included. Two other compounds, skatole and indole, are produced from the amino acid tryptophan in the colon and are absorbed into the blood stream before being degraded in the liver [10]. While indole is thought to be less important with regards to boar taint [11], skatole is found to have a strong faecal and naphthalene taste and odor [12]. Skatole is shown to have medium/high heritability (0.37-0.41) in Norwegian Landrace and Duroc [3]. Levels of skatole are affected by feeding and recently Rasmussen et al. [13] demonstrated that feed containing chicory root could impact the activity of enzymes involved in skatole metabolism.

Genome scans have been performed for androstenone and skatole in a variety of pig breeds [14-17], however results have been inconsistent. Recent developments in high-throughput genotyping technologies and efficient methods for SNP-discovery using next-generation sequencing technologies have paved the way to create efficient tools for QTL mapping by genome-wide association. A high-density porcine SNP array containing more than 60,000 SNPs has been developed [18], which offers a much higher resolution compared to the previously available porcine SNP chip [19] used in our populations [3]. The aim of this study was to perform a high-density genome wide association (GWA) in combination with linkage disequilibrium-linkage analysis (LDLA) using the 60 K SNP chip to identify the genomic regions influencing the boar taint compounds androstenone, skatole and indole, as well as the fertility related compounds 17β-estradiol, estron sulphate and testosterone in the two commercial breeds Duroc and Landrace. Additionally, differentially expressed genes within the QTL regions were detected by combining the current results with the results from Moe et al. [20] and [21]. This identifies genes located within the target regions providing both transcriptional and positional candidate genes for the QTL.

## Methods

### Animals and phenotypes

In total, data from 1,251 purebred Norwegian Landrace and 918 purebred Duroc male pigs tested in NORSVIN's three boar testing stations were included in this study. This includes 92 half sib families with 7 to 24 offspring for Landrace and 70 half sib families with 6 to 27 offspring for Duroc. Pedigree information, including six generations of parents, was available for all individuals. Animals were reared under similar conditions using standard commercial feed, and were sacrificed over a period of 26 months. On average, Landrace and Duroc boars reached slaughter weight (100 kg) at 143 and 156 days respectively, and were slaughtered in average 15 days later. The reason for this is that we had to wait for final EBV estimation and selection of the AI boars. Blood samples were collected immediately before slaughter for plasma suspension and DNA extraction, while subcutaneous adipose tissue samples for androstenone measurement were collected from the neck at slaughter line (20-30 minutes post-mortem). All animals were cared for according to laws, internationally recognized guidelines and regulations controlling experiments with live animals in Norway (The Animal Protection Act of December 20th, 1974, and the Animal Protection Ordinance Concerning Experiments with Animals of January 15th, 1996); according to the rules given by Norwegian Animal Research Authority.

Levels of androstenone were measured in both subcutaneous fat and plasma and in both cases the concentration of androstenone (ppm) was determined using a modified time-resolved fluoroimmunoassay [22], employing an antibody produced by Andresen [23]. Skatole and indole levels in adipose tissue were analyzed using high performance liquid chromatography [24]. Plasma levels of testosterone were measured using a radioimmunoassay (Orion Diagnostica, Espoo, Finland) and displayed intra- and total-assay coefficients of variation (CVs) were 7% and 9%, respectively. Plasma levels of 17β-estradiol were measured using a fluoroimmunoassay (Perkin Elmer, Turku, Finland) producing intra- and total-assay CVs of 3% and 7%, respectively. Plasma levels of estrone sulphate were measured with a radioimmunoassay (Diagnostic System Laboratories, Inc., Webster, TX, USA) producing intra- and total-assay CVs of 5% and 7%, respectively. All chemical compounds are reported in parts-per-million (ppm). All the phenotypic data were log-transformed in order to make them approximately normally distributed.

### DNA extraction, genotyping, quality control and map construction

DNA was extracted from porcine blood, leukocytes or semen using the MagAttract DNA Blood Midi M48 protocol on the Bio-Robot M48 (Qiagen, Hilden, Germany). DNA concentration and quality were assessed using a NanoDrop ND-1000 spectrophotometer (NanoDrop Technologies, DE, USA) and a Victor^3 ^Multilabel Counter (model 1420, Turku, Finland) using PicoGreen reagent (Molecular Probes, OR, USA). The DNA quality was considered to be acceptable with lower and upper limits of 25 and 75 ng/μl, respectively, and with 260:280 and 260:230 ratios around 1.8. Thereafter samples were normalized to 50 ng/μl in 96-well plates. Genotyping of the 60 K porcine SNP array was performed using the iScan platform (Illumina, San Diego, CA, USA) according to manufacturer's instructions. Clustering and genotype calling were performed using the genotyping module in the Genome Studio software (Illumina, San Diego, CA, USA). In total, the 1,251 Landrace and 918 Duroc boars were genotyped for 60,451 SNPs. Genotypes were included in analysis if SNP markers passed a quality threshold of having a minor allele frequency (MAF) > 0.01, call frequency > 0.10, and Parent-Child Mendelian errors < 0.025. Samples were included in analysis if their call rate was > 75%, although the average call rate was 99.4% with a standard deviation of 1.8%. Based on data from a previous study, samples from animals displaying excessive pedigree errors were not included in this analysis. After quality control 1,155 boars and 86 sires were available for analysis in Landrace together with 840 boars and 68 sires in Duroc. In total 51,943 SNP markers (86%) passed the quality threshold filtering. Due to being non-informative 7117 SNPs were removed. Additionally, 346 SNPs were removed since they were not able to place on any of the chromosomes using two-point analysis in a modified version of the CriMap software package [25]. The order of markers was determined based on the porcine sequence Build 9 (Sanger Institute) followed by multipoint linkage analyses using CriMap [25]. Recombination units were then transformed to map distances using the Haldane mapping function, and the suggested positions from Sscrofa9 were modified if required. In total 44,480 of the high quality SNP markers were placed on chromosomal locations on Sscrofa9, 38,396 on the Landrace map and 36,869 on the Duroc map. Number of SNP markers per chromosome and length of the chromosomes (bp and cM) are shown in Additional file 1.

### Statistical analyses

Statistical analyses were performed separately for the two breeds. Preliminary estimates of the phenotypes were found to have a skewed distribution and were consequently log-transformed. Furthermore some animals had phenotypic levels below the detection limits of the chemo analytical methods used and were recorded as zero. To avoid losing information caused by log-transformation, the detection limits were added to all phenotypes before transformation. The detection limits were 0.05 ppm for androstenone, 0.01 ppm for skatole and indole, 0.5 ppm for testosterone and estron sulphate, and 0.04 ppm for 17β-estradiol. GWA is considered to be more powerful than linkage analysis for detecting the effects of common alleles with small effects but is less powerful when traits have a complex genetic determination, including epistasis. The GWA method uses linkage disequilibrium information and assumes all markers to be independent of each other, thereby ignoring genetic linkage between markers. GWA is expected to reveal more false positive results because single marker linkage disequilibria are very variable. Therefore, the data was simultaneously analyzed with LDLA, combining the linkage disequilibrium and linkage analysis [26].

#### Genome-wide association study (GWAS)

A GWAS was conducted using a statistical model including fixed effect corrections for; test-station, age at entering the test, number of days in test, number of litter mates born alive, and the random effects; a polygenic effect and an additive SNP effect. The polygenic effect was included to account for the family structure that was present in the data and was assumed to have a covariance matrix proportional to the pedigree based relationship matrix. The additive SNP effect was also assumed a random effect, and was obtained by regressing the phenotypes on to the number of '1' alleles in the SNP genotypes, i.e. for the SNP genotypes '0 0', '0 1', and '1 1' the covariate of the regression was 0, 1 and 2, respectively, where arbitrarily one of the SNP alleles is called '1' and the other '0'.

A log-likelihood ratio test-statistic was calculated as LnLikratio = LnLik_model incl. SNP _- LnLik_model excl. SNP_, where LnLik_model incl. SNP _is the REML log-likelihood [27] of the model describe above and LnLik_model excl. SNP _is the REML log-likelihood of the same model, except that the SNP effect was excluded from the analysis. Under the null-hypothesis of no SNP effect, 2*LnLikratio was assumed to follow a chi-squared distribution with one degree of freedom. Chromosome-wide P-values (CWS) were obtained using the approach described by Piepho [28]. Genome-wide P-values (GWS) were obtained by multiplying the CWS by 18, i.e. by the number of porcine autosomal chromosomes analyzed. We considered the SNP/QTL as significant if P < 0.05.

#### Linkage disequilibrium/linkage analysis (LDLA)

The statistical model used for the combined linkage disequilibrium and linkage analysis (LDLA) was identical to that of GWAS except that a haplotype effect was fitted instead of a SNP effect. LDLA followed approximately the approach of Meuwissen and Goddard [26], except that haplotypes were either assumed to be completely correlated or uncorrelated, instead of fitting a more differentiating IBD matrix G. Based on experiences with IBD matrix calculations it was decided that if the sum of the number of identical SNP alleles equal to the left and to the right of the putative QTL position before a non-equal allele occurred was ≥ 10, then a haplotype pair was considered identical. Otherwise, the two haplotypes were considered to be different, and uncorrelated. LnLikratio, CWS and GWS were calculated in the same way as for GWAS.

### Candidate genes and differential expressed genes within QTL regions

Candidate genes within the QTL regions were detected using the porcine Ensembl data base (http://www.ensembl.org/Sus_scrofa/Info/Index) (Build 9). In order to obtain additional candidate genes in comparative genomic regions the UCSC genome browser (http://genome.ucsc.edu/index.html) and the NCBI database (Build37) were applied. To find differentially expressed genes within the significant QTL regions, the results from the current QTL study was compared with the significant differentially expressed genes obtained using the same animal material [20,21]. The results were imported and combined in R v.2.9.2 [29].

## Results

Descriptive data (number of animals, overall means and standard deviations of log-transformed data, and max/min values) for this animal material have been presented earlier by Grindflek et al. [3]. Here we present QTL results from the seven traits; androstenone in subcutaneous fat (AndroF), androstenone in plasma (AndroP), skatole (Skat), indole (Indo), testosterone (Testo), 17β-estradiol (Ediol) and estronsulphate (Esulph).

### Androstenone and other sex steroids

**Figure 1:**
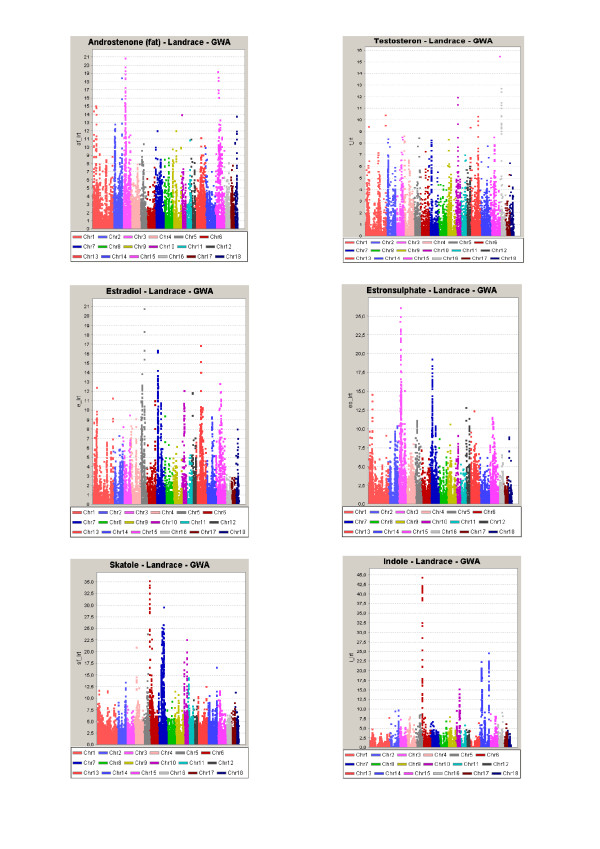
**Log-ratio plots for AndroF, Testo, Ediol, Esulph, Skat and Indo from the genome wide association study (GWAS) in Landrace**.

**Figure 2:**
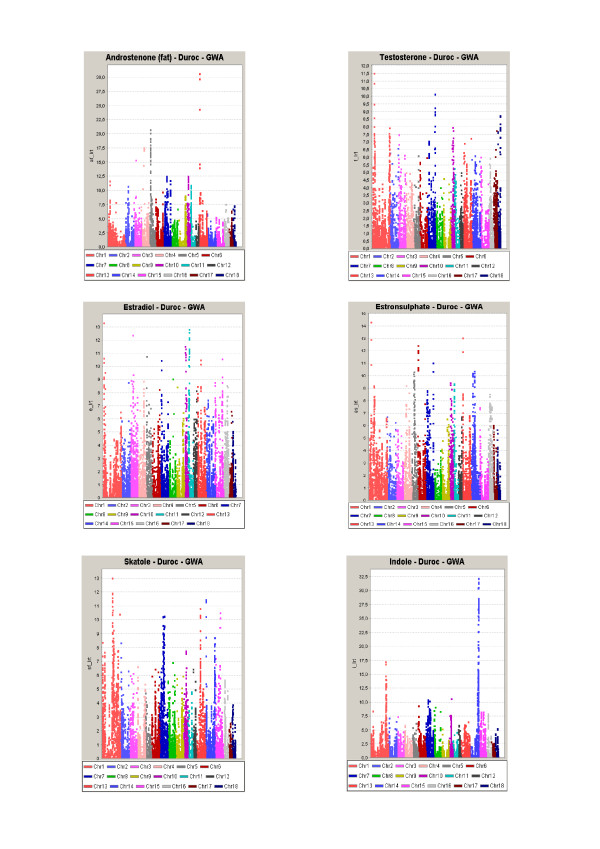
**Log-ratio plots for AndroF, Testo, Ediol, Esulph, Skat and Indo from the genome wide association study (GWAS) in Duroc**.

According to the threshold values described in Material and Methods we detected 14 GWS QTL regions for AndroF in Landrace, and 14 GWS QTL regions in Duroc using LDLA. Seven of these QTL regions were detected in both breeds, in every case they were significant (P < 0.05) at the GWS level in one breed and at least CWS in the other (P < 0.05). The common QTLs were located at SSC 1, 2, 3, 7 and 15. Results from the GWAS revealed 10 QTL regions for AndroF that were GWS in Landrace, 7 of these were located within QTL regions detected by LDLA. In Duroc, 11 QTLs were detected with GWAS, 10 falling within QTL regions identified with LDLA. For AndroP, 14 and 8 GWS QTLs were detected in Landrace and Duroc (respectively) using LDLA (Table 1), with 7 and 5 of them confirmed using GWAS, respectively (Figure 1 and 2). When comparing the QTL regions detected by LDLA, we found that 12 QTLs are significant for both AndroF and AndroP in Landrace, and 4 in Duroc. Comparing QTL regions at the CWS level, a greater number of QTL regions are shared between the AndroF and AndroP. All 14 GWS AndroF QTLs in Landrace are also affecting Ediol and/or Esulph on at least a CWS level. In Duroc, 3 of 14 QTLs are affecting AndroF without at least CWS affecting any of the other estrogens. Only 2 GWS QTLs were detected for Testo (SSC10, 28-37 Mb, and SSC16, 21-24 Mb), however these were very convincingly obtained in both breeds using both LDLA and GWAS. For Ediol, 13 GWS QTLs were obtained in the LDLA for Landrace and 6 for Duroc, with the common QTLs between breeds located on SSC 1, 13 and 15. QTL regions for Esulph were generally similar to those detected for Ediol. For Landrace, 10 of the 13 QTLs obtained in Ediol were also at least CWS in Esulph. Notably however, a convincing QTL on SSC3 (44.5-44.6 Mb) was significant (GWS) in Esulph only. In Duroc all QTLs obtained on GWS level were in common for Ediol and Esulph. The fraction of the genetic variance explained by the significant AndroF QTL regions varies between 1 and 6% (results not shown). The GWS QTLs for AndroF obtained by LDLA are listed in Table 1. The log-ratio plots from GWAS results AndroF, Testo, Ediol and Esulph are shown in Figure 1 and 2 for Landrace and Duroc, respectively. The candidate genes within GWS QTL regions for AndroF are shown in Table 2 and the differentially expressed genes in high/low androstenone boars located within these QTL regions are shown in Table 3.

**Table 1:** The genome significant QTLs (from LDLA) for androstenone in fat (AndroF)

SSC	QTL	Breed	LnLikratio	CWS_P**	GWS_P**	Mb_Conf.Int.^$^
1	1a	L*/D*	11.57/5.87	0.0014	0.025	15.2-16.3
1	1b	D*	11.67	0.0006	0.011	33.0-33.4
1	1c	L*	15.07	0.0002	0.004	44.3-44.9
1	1d	L*	11.73	0.0013	0.023	53.5 - 54.3
2	2a	L*/D*	12.73/9.06	0.0016	0.029	29.9 - 37.0
2	2b	L*/D	9.11/21.12	< 0.0001	< 0.001	101.4 - 101.7
2	2c	L*	12.32	0.0020	0.036	132.8 - 135.9
3	3a	L*/D*	14.19/11.07	0.0003	0.005	32.2-53.6
3	3b	L*	18.73	< 0.0001	< 0.001	107.0 - 107.1
4	4	D	17.52	< 0.0001	< 0.001	47.5 - 48.0
5	5a	D	20.74	< 0.0001	< 0.001	20.4 - 22.2
5	5b	L*	10.23	0.0014	0.025	32.4 - 34.9
6	6	D*	9.79	0.0018	0.032	118.8 - 119.2
7	7a	L*/D*	7.37/9.10	0.0010	0.018	34.6-40.2
7	7b	L/D*	4.59/12.36	0.0006	0.011	62.5 - 65.0
7	7c	D*	11.80	0.0008	0.014	126.9 - 127.5
9	9	L*	12.01	0.0005	0.009	7.5-8.0
10	10	D*	12.55	0.0004	0.007	35.0 - 37.0
11	11a	D*	10.67	0.0011	0.020	11.8 - 16.7
11	11b	L*	12.31	0.0005	0.009	64.2 - 64.5
13	13a	D*	30.61	< 0.0001	< 0.001	19.3 - 19.6
13	13b	L*	13.34	0.0003	0.005	87.8 - 92.5
15	15	L*/D*	14.80/4.85	0.0003	0.008	42.5 - 70.7
18	18a	L*	10.66	0.0011	0.020	13.4 - 14.2
18	18b	L*	12.26	0.0005	0.009	39.7 - 40.4

* Additionally at least Chromosome Wide Significant (CWS) for Ediol and/or Esulph

** The highest P-value from the two breeds

$ The boundaries of the 90%-confidence-interval in mega-bases (Mb).

**Table 2:** Candidate genes located within GWS QTL regions and discussed in the paper

QTL	Positions (Sscrofa9)	Reference	Full name	Gene
2a	41501759 - 41519677	De Fabiani et al., 2010	Cytochrome P450, subfamily IIR, polypeptide 1	CYP2R1
2b	10440795-104856025	Rodriquez-Agudo et al., 2008	START domain containing 4, sterol regulated	STARD4
3a	39059355 - 39072049	Gumus et al., 2008	Interleukin Alpha 1	IL1A
3a	42095144 - 42118620	Horibata & Sugimoto, 2010	START domain containing 7, sterol regulated	STARD7
3a	44005314 - 44096991	Bray et al., 2009	UDP-glucuronate decarboxylase 1	UXS1
4	47901914-47937394	Amills et al., 2005	2,4-alpha-dienoyl-CoA reductase 1	DECR1
4	47870808-47893791	Opperman et al., 1992	Calbindin 1, 28kDa	CALB1
5	hsa12: 56114151-56118525*	Wang et al. 1999	Retinol dehydrogenase	Rdh5
5	hsa12: 57157108- 57181574*	Moeller & Adamski, 2006	Hydroxysteroid (17-beta) dehydrogenase 6 homolog	HSD17B6
7a	27908299 - 27911241	Crawford et al., 1992	cytochrome P450, fam. 21,subfam. A, polypeptide 2 iso a	CYP21/CYP21A2
7b	64454785 - 64461043	Diaz & Squires, 2000	cytochrome P450, family 1, subfamily A, polypeptide 1	CYP1A1
7b	hsa15:74630100 - 74660081*	Miller, 1995	cytochrome P450, family 11, subfamily A, polypeptide 1	CYP11A1
7c	127029340-127072433	Wiebe et al., 2010	vaccinia related kinase 1	VRK1
7a^s^	64280270 - 64285232	Lanza and Yost, 2001	cytochrome P450, family 1, subfamily A, polypeptide 2	CYP1A2
13	19112438 - 19141042	Bahn et al., 2005	solute carrier family 22, member 14	SLC22A14
13	19146069 - 19153361	Bahn et al., 2005	solute carrier family 22, member 13	SLC22A13
14	148132846-148145308	Babol et al., 1998	cytochrome P450, family 2, subfamily E, polypeptide 1	CYP2E1
15	64064865-64074555	Christoffersen et al., 2010	Glucagon	GCG
15	hsa8: 38000226-38008783*	Christenson & Strauss, 2000	Steroidogenic acute regulatory protein	STAR

* Gives the comparative human genomic positions on Build37 for the genes not annotated in Sscrofa9.

**Table 3:** Differentially expressed genes within GWS QTL regions

Gene	Full name	SSC positions (Sscrofa9)	QTL	Breed**	DE tissue
C6orf211	chromosome 6 open reading frame 211	1: 15,909,144-15,924,522	1a	D	Testis
GPD1L	glycerol-3-phosphate dehydrogenase 1-like	1: 44,341,710-44,342,765	1c	L	Testis
GAS2	growth arrest-specific 2	hsa11: 22,647,188-22,834,601*	2a	L	Testis
DCI	dodecenoyl-CoA isomerase	hsa16: 2,289,396-2,301,615*	3a	D	Testis
GLIS2	GLIS family zinc finger 2	hsa16: 4,364,762-4,389,598*	3a	D	Liver, testis
TIGD7	tigger transposable element derived 7	3: 34,867,636-34,868,997	3a	L	Testis
RAB11FIP3	RAB11 family interacting protein 3 (class II)	3: 35,607,368-35,662,467	3a	D	Testis
NARFL	nuclear prelamin A recognition factor-like	3: 35,702,474-35,708,696	3a	D	Liver
GNPTG	N-acetylglucosamine-1-phosphate transferase, gamma subunit	3: 36,242,649-36,244,181	3a	L	Testis
IL1A	interleukin 1, alpha	3: 39,059,355-39,072,049	3a	D	Testis
IL1R1	interleukin 1 receptor, type I	3: 47,896,450-47,909,993	3a	D	Liver
CALB1	calbindin 1, 28kDa	4: 47,870,808-47,893,791	4	D	Testis
SCUBE3	signal peptide, CUB domain, EGF-like 3	7: 35,596,571-35,617,151	7a	L	Testis
C6orf89	chromosome 6 open reading frame 89	7: 37,180,914-37,212,104	7a	D	Testis
PTPN9	protein tyrosine phosphatase, non-receptor type 9	7: 63,302,924-63,419,418	7b	D	Testis
CYP11A1	cytochrome P450, family 11, subfamily A, polypeptide 1	hsa15: 74,630,100-74,660,081*	7b	D, L	Testis
EXOSC8	exosome component 8	11: 12,214,101-12,412,285	11a	D	Testis
KCNMB2	potassium large conductance calcium-activated channel, subfam M, b memb 2	13: 89,166,152-89,194,414	13b	L	Liver
MLF1IP	MLF1 interacting protein	15: 42,999,826-43,027,571	15	D	Testis
SORBS2	sorbin and SH3 domain containing 2	15: 43,835,332-44,063,424	15	L	Liver
TM2D2	TM2 domain containing 2	15: 44,964,565-44,971,596	15	L	Liver
RBPMS	RNA binding protein with multiple splicing	15: 51,472,724-51,785,193	15	D	Testis
FMNL2	formin-like 2	15: 56,320,296-56,433,061	15	L	Testis
ARL6IP6	ADP-ribosylation-like factor 6 interacting protein 6	15: 56,490,709-56,522,096	15	L	Liver, testis
CD302	CD302 molecule	15: 62,141,546-62,172,624	15	D	Testis
GCG	glucagon	15: 64,064,865-64,074,555	15	L	Testis
RND3	Rho family GTPase 3	hsa2: 151,324,709-151,395,525*	15	L	Liver
STAR	steroidogenic acute regulatory protein	hsa8: 38,000,226-38,008,783*	15	L	Testis
TNFAIP6	tumor necrosis factor, alpha-induced protein 6	hsa2: 152,214,106-152,236,560*	15	L	Testis
ZRANB3	zinc finger, RAN-binding domain containing 3	hsa2: 135,894,486-136,288,806*	15	D	Testis

Gene expression data was obtained from Moe et al. [20,21].

* Gives the comparative human genomic positions on Build37 for the genes not annotated in Sscrofa9.

** Breeds are L = Norwegian Landrace and D = Norwegian Duroc.

### Skatole and indole

For skatole, 10 and 4 QTLs were GWS in the LDLA analysis for Landrace and Duroc, respectively, 8 and 4 of them (respectively) were also GWS using GWAS. Four QTLs were obtained in both breeds using the LDLA, on at least CWS level. In 7 of the 10 GWS LDLA QTLs obtained in Landrace, the QTLs were also affecting (at least CWS) the level of Indo. Likewise, 3 of 4 GWS Skat QTLs in Duroc were also significantly affecting Indo. The GWS QTLs for Skat obtained by LDLA are located at SSC 1, 5, 6, 7, 10, 11, 13 and 14, and are all listed in Table 4. Log-ratio plots of GWAS results for Skat and Indo are presented in Figure 1 and 2 for Landrace and Duroc, respectively. The candidate genes within GWS QTL regions for Skat are shown in Table 2.

**Table 4:** The genome significant QTLs (from LDLA) for skatole

SSC	QTL	Breed	LnLikratio	CWS_P**	GWS_P**	Mb_Conf.Int.^$^
1	1a^s^	L*/D	5.56/13.0	0.0003	0.005	165.5 - 170.1
5	5^s^	L*	11.45	0.0007	0.013	63.2 - 68.4
6	6a^s^	L*	36.30	< 0.0001	< 0.001	3.7-5.0
6	6b^s^	L*	16.50	< 0.0001	< 0.001	32.2 - 39.4
7	7a^s^	L/D*	27.98/10.25	< 0.0001	< 0.001	61.5 - 69.6
7	7b^s^	L/D*	24.29/10.25	< 0.0001	< 0.001	75.9-81.6
10	10a^s^	L*	10.83	0.0011	0.018	11.3 - 13.6
10	10b^s^	L*	14.52	0.0001	0.002	53.1-55.3
11	11^s^	L	9.88	0.0017	0.030	15.7 - 16.7
13	13^s^	D*	10.81	0.0010	0.018	60.5 - 65.6
14	14a^s^	L*	10.34	0.0017	0.031	38.1 - 39.1
14	14b^s^	L*/D*	9.76/8.27	0.0024	0.042	146.4 - 148.4

* Additionally at least Chromosome Wide Significant for indole

** The highest P-value from the two breeds

^s ^All QTLs for skatole are marked with s.

$ The boundaries of the 90%-confidence-interval in mega-bases (Mb).

## Discussion

### QTL regions and potential candidate genes

The GWS QTLs for androstenone and skatole obtained by LDLA are divided into the different chromosomes and discussed below. We chose to focus on the LDLA results since this method is considered to be most robust. Moreover, the LDLA likelihood profiles were smoother than those from GWA which facilitated the positioning of the QTLs. The QTLs detected by LDLA are, however, highly supported by the GWAS results obtained in the study (Figures 1 and 2). Candidate genes located within QTL regions and discussed in the paper are all listed with the full name in Table 2.

### SSC1

#### Androstenone

Four putative QTLs were detected for AndroF in Landrace and/or Duroc on SSC1 (positions shown in Table 1). Recently on SSC1, three QTLs were also detected in a Dutch composite Duroc-line [17] and three QTLs in French Large White [30], but all in different regions. No obvious candidate genes were observed in any of the regions detected in our study, however a non-annotated gene transcript (*C6orf211*) located within QTL1a was previously found to be differentially expressed in Duroc boars with extreme high/low androstenone levels, and the gene *GPD1L *located within QTL1c was up-regulated in high androstenone Landrace boars [20] (Table 3).

#### Skatole

One putative QTL for skatole was detected on SSC1 position 165.5-170.1 Mb (Table 4). This QTL did not overlap with either of the two recently detected QTL positions on SSC1 for skatole in French Large White [30]. The peak positions in French Large-White are 109.5 and 288.75 Mb, quite far from our QTL at 165-170 Mb, and are most likely two different QTLs not segregating in our Landrace and Duroc populations.

### SSC2

Two regions on SSC2 (29.9-37.0 Mb; QTL2a, and 101.4-101.7 Mb; QTL2b) are affecting (GWS) AndroF, AndroP, and one or two of the estrogens in both breeds. QTL2a is additionally CWS for Testo in Landrace. A Landrace specific QTL (2c) in region 132.8-135.9 Mb was found to affect all traits included in this study (at least CWS level). Our QTL2a concurs with a wide QTL region for androstenone detected between markers SW240 (18 Mb) and S0226 (48 Mb) in a Large White × Meishan cross [15]. A large number of genes are embraced by this region including *GAS2 *(Table 3) which was previously found to be differentially expressed in testis from Landrace boars with extreme high/low levels of androstenone [20]. One obvious candidate in the region is the *CYP2R1 *gene (Table 2) which encodes a member of the cytochrome P450 superfamily of enzymes known to catalyze many reactions involved in synthesis of cholesterol, steroids and other lipids (e.g. reviewed by De Fabiani et al. [31]). No QTLs for androstenone have previously been reported close to our QTL2b. Despite QTL2b presenting a narrow QTL region we found several candidate genes that may be involved in processes of androstenone metabolism. One of them is the *STARD4 *gene which plays an important role as a directional cholesterol transporter in the maintenance of cellular cholesterol homeostasis [32], and is required to maintain optimum steroid synthesis [33].

### SSC3

In SSC3 we obtained two GWS QTL regions affecting AndroF and AndroP. QTL3a embraces the region 32.2-53.6 Mb in Landrace and 38-40 Mb in Duroc, while QTL3b was detected in Landrace only and is positioned around 107 Mb. Support for QTL3a is provided by Quintanilla et al. [14] who reported a QTL between the markers SW487-S0372 affecting androstenone at different ages in a Large-White × Meishan cross. We found that the QTL3a is also affecting estrogens in both breeds, and that the Landrace specific QTL3b affects both testosterone (results not shown) and estrogens (Table 1). The genes *DCI*, *GLIS2*, *TIGD7*, *RAB11FIP3*, *NARFL*, *GNPTG*, *IL1A *and *IL1R1 *are all localized within QTL3a (Table 3) and found to be differentially expressed in testis or liver in high androstenone Landrace and/or Duroc [20,21]. Interestingly, *IL1A *has been found to interact with *CYP1A1 *[34], which is localized within the QTL7b peak (see Table 1). *CYP1A1 *has previously been proposed as a candidate gene for skatole [35], but is also recently shown to be involved in steroidogenesis [36]. Another candidate in the QTL3a is *STARD7*, which is involved in cellular cholesterol homeostasis [37].

Using Haploview [38] we detected a highly conserved region (43-45 Mb) in QTL3a containing an interesting candidate gene *UXS1 *(at 44 Mb). This gene encodes enzymes catalyzing the formation of progesterone, is involved in the formation of UDP-xylose, and influences 3-beta-hydroxy-delta5-steroid dehydrogenase (HSD3B) activity [39]. It is well known in the boar taint research that the HSD3B enzymes are essential for the biosynthesis of all active steroid hormones [40]. Additionally, *UXS1 *is shown to be differentially expressed after induction of anterior pituitary hormone (ACTH), an important developmental signal that accelerates the appearance of a more mature phenotype in the adrenal glands of young individuals [41].

### SSC4

On SSC4 a QTL for AndroF and AndroP was detected in Duroc only at a position around 48 Mb. This QTL is not affecting any of the other sex hormones and might therefore be very interesting for selection purposes. Other QTLs have previously been detected on SSC4, although they are all located to other chromosomal positions [14,15]. The *DECR1 *gene is located within the narrow QTL position detected in the current study. DECR1, a nuclear encoded mitochondrial enzyme that participates in the *β*-oxidation pathway, has previously been suggested as a candidate involved in several meat quality traits [42]. Furthermore, the candidate gene *CALB1 *(around 47.9 Mb), was found to be differentially expressed in testis from Duroc with extreme high/low levels of androstenone [20]. Interestingly, results suggest that *CALB1 *is regulated by estrogens [43] and other steroid hormones and is through this probably influencing the sexual development and function [44].

### SSC5

#### Androstenone

**Figure 3:**
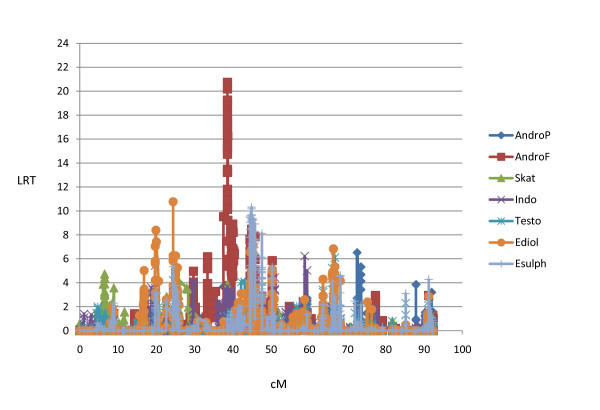
**Likelihood ratio (LRT) profile from LDLA for SSC5 in Duroc**.

A highly convincing GWS Duroc specific QTL for AndroF was detected on SSC5 (QTL5a; 20.4-22.2Mb, Figure 3). Comparative genomic alignment to human sequence revealed that the *hydroxysteroid (17-beta) dehydrogenase *6 homolog (*HSD17B6*), which is known to be involved in steroid metabolism [45], is expected to be located within this region. Additionally, the gene encoding retinol dehydrogenase (*Rdh5*), which is found to be involved in hydroxysteroid dehydrogenase activity by recognizing 5alpha-androstan-3alpha, 17beta-diol and androsterone as substrates [46], is expected to be localized within this QTL region. With respect to implementation in breeding schemes, it is very interesting to note that this QTL does not affect any of the other sex related hormones in this study. Furthermore, a Landrace specific QTL (5b) affecting AndroF, AndroP and the other sex hormones was detected in position 32.4-34.9 Mb. No QTL for androstenone have previously been mapped to SSC5.

#### Skatole

A QTL affecting Skat and Indo was detected in Landrace in the region 63.2-68.4 Mb (Table 4). In exactly the same chromosomal region we also obtained GWS for AndroP and Ediol, and CWS for Testo and Esulph (results not shown). This is a rather gene rich region but no obvious candidate genes were found.

### SSC6

#### Androstenone

Recently, Duijvesteijn et al. [17] reported a cluster of 31 SNPs on SSC6 (36.9-44.9 Mb) significantly affecting subcutaneous fat androstenone in a Dutch Duroc sire-line. Interestingly, in our study a CWS QTL region affecting both AndroF and AndroP was detected between 35.7 and 42.8 Mb in Duroc, but not in Landrace. This QTL does not significantly affect (P < 0.05) the other sex hormones included in this study, although the effect on Esulph is very close to significant. A large block of strong linkage disequilibrium (LD) was detected between 36.9 and 40 Mb in the Dutch population [17], which is noted to be a very gene rich region. In our study we also see a highly extended amount of LD on approximately the same level as the Dutch study in the region between 35.9 and 39.3 Mb (results not shown). However, the most significant haplotype in the QTL region, 13 SNPs in complete LD between 38-39 Mb, are explaining only 1.2% of the total genetic variation. In our study a Duroc specific GWS QTL was located in the vicinity of 119 Mb (Table 1) which affects AndroF at GWS level and AndroP, Testo, Ediol and Esulph at CWS level.

#### Skatole

**Figure 4:**
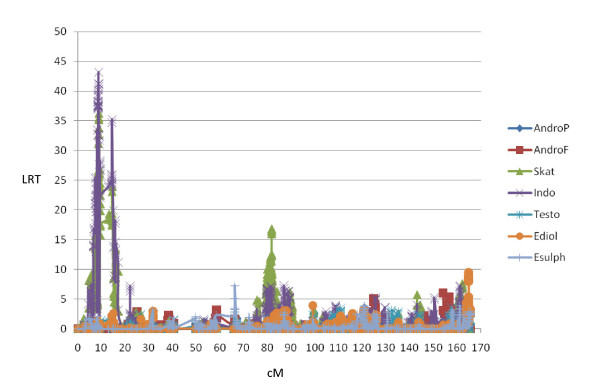
**Likelihood ratio (LRT) profile from LDLA for SSC6 in Landrace**.

A Landrace specific QTL for Skat and Indo was obtained on SSC6, region 3.7-5.0 Mb (QTL6a^s^), and demonstrated the highest log-ratio found in this study (Table 4 and Figure 4). Searching for genes in porcine and comparative human genomes using Ensembl failed to reveal any obvious candidates, although several transcription factors are located within this region. Interestingly, a QTL for 'sensory panel skatole' has previously been detected in a Meishan/Large White cross located close to marker SW1353 (approximately position 7.0 Mb) in a very low density marker map (Lee et al. [15]). Furthermore, a GWS QTL (6b^s^) for Skat and Indo is obtained between position 35.9-39.4 Mb (Table 4), which is within the same region as the CWS AndroF QTL described in Duroc above and recently detected by Duijvesteijn et al. [17]. This is a very gene rich region with several biologically relevant genes.

### SSC7

#### Androstenone

A large region of highly significant QTL-effects for androstenone was found on SSC7 between positions 33.6 and 88.3 Mb in both breeds. This result is supported by previous studies covering similarly large overlapping QTL regions between markers SW1354 and SW632 [14] and between markers TNFB (position 27.7 Mb in Sscrofa9) and S0066 (position 56.8 Mb in Sscrofa9) [15]. Our results, however, seem to detect three different QTLs within the region, localized at 33.6-41.9 Mb (QTL7a), 52.8-75.1 Mb (QTL7b) and 80.8-88.3 Mb (QTL7c) (Table 1). Differentiation of these three closely positioned QTLs is made possible by the improved marker density in this study and therefore finer mapping resolution. In addition to the QTLs 7a, 7b and 7c for AndroF, we detect a GWS QTL for AndroP, Testo, Ediol and Esulph (results not shown) in position 27.4-27.9 Mb which embraces the candidate gene *CYP21 *(or *CYP21A2*). CYP21 is a member of the cytochrome P450 superfamily enzymes, which is a key enzyme for corticosteroidogenesis [47] and suggested to have arisen evolutionary from the same gene as *CYP17A1 *[48]. Physiologically, CYP21 is known to lead to drastic fertility changes in human females [49], and *CYP21 *has been found to be differentially expressed in Landrace testes with high/low androstenone levels [50]. It is also relevant to note that a QTL for age-at-puberty has been detected in the same region, across a 24 cM interval around marker TNF (position 27.7 Mb in Sscrofa9; [51]), and we know that at least estron sulphate (Esulph) is well known to be correlated with sexual maturity [8]. Two genes located within QTL7a, *SCUBE3 *and *C6orf89 *(Table 3), are differentially expressed in testis from Landrace and Duroc boars with high/low levels of androstenone, respectively [20]. In addition to being GWS for AndroF and AndroP, and CWS for Testo, Ediol and Esulph, QTL7b is highly GWS for skatole and indole in both breeds (see below). The gene encoding *CYP1A1 *is located within this region and is involved not only in skatole metabolism [52], but also in steroidogenesis [36]. From comparative mapping with humans, another gene (*CYP11A1*) is predicted to be located close to *CYP1A1*. The CYP11A1 enzyme is known to catalyze the conversion of cholesterol to pregnenolone in the first and rate-limiting step of the steroid hormone synthesis [53]. Previous results from the same Norwegian populations used in this paper show differential expression of *CYP11A1 *in the liver of high-androstenone boars, however no significant association was detected in Landrace, between a *CYP11A1 *SNP and androstenone [50]. Similarly, no significant *CYP11A1 *SNP associations with androstenone were detected in the Large White and Meishan cross performed by Quintanilla et al. [14], which may be evidence to suggest that another candidate gene(s) is involved and explains the differences seen for androstenone level. The gene *PTPN9 *localized within the QTL7b (Table 3) has previously been found to be up-regulated in high androstenone Duroc testis [20]. Only one gene (*VRK1*; Table 2) has been annotated in QTL7c (126.9-127.5 Mb). This gene has a role in regulating gametogenesis, and disrupted VRK1 function is shown to result in infertile male and female mice [54].

#### Skatole

**Figure 5:**
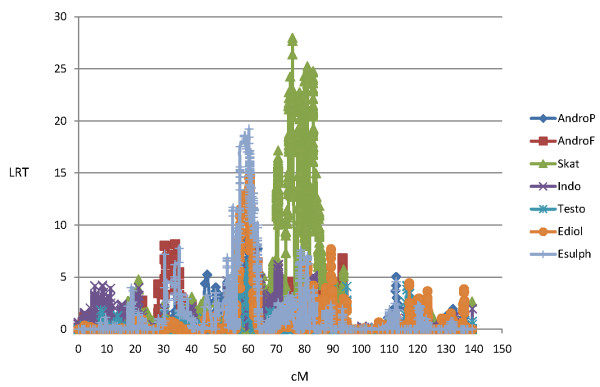
**Likelihood ratio (LRT) profile from LDLA for SSC7 in Landrace**.

QTL7a^s ^for Skat is located between 61.5 and 69.6 Mb and therefore overlaps with the QTL7b for androstenone discussed above. The *CYP1A1 *and *CYP1A2 *genes are oriented head-to-head in QTL7a^s ^and the enzyme products are found to be involved in degradation of skatole [13,52]. Additionally, we detected a highly GWS QTL (QTL7b^s^) affecting Skat and Indo in both breeds (Table 4). The LRT profiles for Landrace, covering the most convincing QTL(s), are shown in Figure 5. QTL for skatole have recently been detected on SSC7 in a French population but in a different region [30].

### SSC9

A Landrace specific QTL affecting AndroF on GWS level and AndroP, Ediol and Esulph on CWS level was detected between 7.5 and 8.0 Mb on SSC9. A QTL for androstenone has previously been detected on SSC9 in a French Large White population [30] and for skatole in a Meishan/Large White cross [15], although these have both been found in different chromosomal regions compared to our study.

### SSC10

#### Androstenone

A Duroc specific QTL was detected on SSC10 affecting androstenone at GWS level and affecting Testo, Ediol, Esulph, Skat and Indo at CWS level. No known genes are located within this region in pigs. It is however notable that a QTL for abnormal odor has been reported in the same chromosomal region (between markers SW1041-SW951) by Lee et al. [15].

#### Skatole

Two GWS QTLs (QTL10a^S ^and QTL10b^S^) for Skat and Indo were detected for Landrace only. No other QTL for skatole have previously been detected on SSC10.

### SSC11

#### Androstenone

Two GWS breed specific QTLs were detected in positions 11.8-16.7 Mb (QTL11a) and 64.2-64.5 Mb (QTIL11b) in Duroc and Landrace, respectively. Both QTL11a and QTL11b were also at least CWS for both Ediol and Esulph. A QTL for androstenone in subcutaneous fat has been detected in French Large White [30] but in another position (37.1-37.8 Mb). In the region of QTL11a it was previously found [20] that transcripts from *EXOSC8 *were differentially expressed in testis from high/low androstenone boars (Table 3).

#### Skatole

A Landrace specific QTL was detected in position 15.7-16.7 Mb affecting Skat but not Indo, in both GWA analysis and LDLA methods. No QTLs for skatole have previously been detected on SSC11.

### SSC13

#### Androstenone

**Figure 6:**
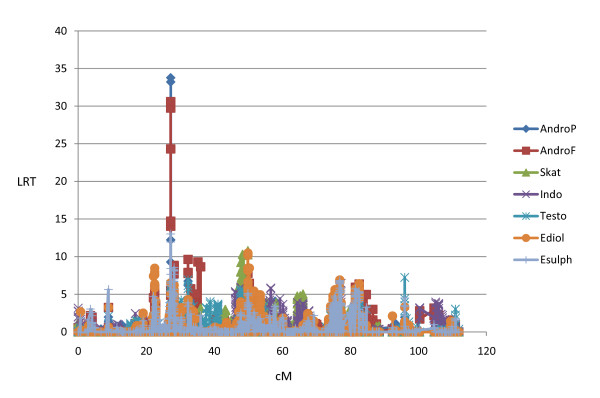
**Likelihood ratio (LRT) profile from LDLA for SSC13 in Duroc**.

A highly significant and narrow region, 19.3-19.6 Mb (QTL13a) was detected in Duroc for both AndroP and AndroF, as well as for the other three sex steroids investigated in this study (Figure 6). A similar region is detected in Landrace, but for AndroP, Ediol and Esulph only (results not shown). No obvious candidate genes are located within the confidence interval, although two candidate genes in the solute carrier family 22, members 13 and 14 (*SLC22A13 *(*OAT*) and *SLC22A14 *(*OAT10*)) are located very close (19.1 Mb). The substrate panel of *SLC22A*s includes important endogenous compounds like tryptophan metabolites [55], and sulfated steroids [56]. A Landrace specific QTL for AndroF and the two estrogens was detected from 87.8-92.5 Mb (QTL13b). Although not exactly corresponding, a QTL for 'androstenone at age of 120 days' was detected in a Large White/Meishan cross close to marker SWR1941 at 74 Mb [14], notably with a much lower marker density. Two other QTLs were detected in a Large White population [30], although at more distant chromosome positions. The gene *KCNMB2*, expected to be located within QTL13b, was previously found to be differentially expressed in liver of high/low androstenone Landrace boars (Table 3). Interestingly, gene expression of *KCNMB2 *is previously shown to be increased by 17β-estrogen stimulation, although this is studied in human Müller cells only [57].

#### Skatole

A Duroc specific QTL for Skat and Indo was detected from 60.5 to 65.6 Mb. One QTL has previously been detected for skatole on SSC13 [30] but once again the position is not in agreement with our results.

### SSC14

Two GWS QTLs for skatole were detected on SSC14, one of these was Landrace specific and extended from 38.1 to 39.1 (QTL14a^S^). No QTL for skatole have previously been observed in this region, although a QTL for 'androstenone at: 100, 120, 140 and 160 days' has been detected in the same region in a Large White/Meishan cross [14]. The second QTL extended between 146.6 and 148.4 Mb (QTL14b^S^) and presented GWS for skatole in Landrace, and CWS (p < 0.05) in Duroc. Furthermore, the QTL is highly GWS for indole in both breeds. The gene encoding cytochrome P450 2E1 (*CYP2E1*), which has a central role in the metabolism of skatole [e.g. 58, 59], is located at 148.1 Mb and is an excellent positional and biological candidate gene for this QTL. A previous study from our group utilizing exactly the same animal material, detected a haplotype within *CYP2E1 *which explained up to 6% of the phenotypic variance for skatole and up to 12% of the phenotypic variance for the indole [60]. Our results are in close agreement with previous findings in Danish Landrace [61].

### SSC15

A very wide GWS QTL located across a confidence interval from 42.5-70.7 Mb on SSC15 in both breeds was affecting all the traits in this study (at least CWS), except for Indo. This is a very gene rich region including several genes involved in sulfotransferase activity belonging to the cytochrome P450 family. From the studies of Moe et al. [20,21] as many as 12 genes within this QTL region were found to be differentially expressed in liver and/or testis in high/low androstenone boars (Table 3). This indicates that several genes might be causing the QTL effect although only the genes encoding *GCG *(glucagon) and *STAR *(steroidogenic acute regulatory protein) are known to be correlated with level of different steroids like androgens and estrogens [50,62,63]. As far as we know no QTLs for boar taint have previously been found on SSC15.

### SSC18

Two Landrace specific QTLs were detected on SSC18 in positions 13.4-14.2 Mb (QTL18a) and 39.7-40.4 Mb (QTL18b). As far as we know no QTLs for boar taint have previously been detected on SSC18.

## Conclusions

This is the first report utilizing a high density marker map for simultaneous analysis of boar taint compounds and related sex hormones, using both GWA and LDLA approach. Several QTLs appear to be involved in regulation of androstenone and skatole, individually they explain relatively little of the total genetic variation. Most of the QTLs for androstenone are affecting both androstenone and estrogens, making practical implementation in breeding challenging. Most QTLs are also breed specific and we conclude that panels of SNPs for selection need to be designed for each breed separately. However, 7 QTLs were detected in one breed and confirmed in the other breed, i.e. an independent sample. QTLs that were not confirmed across breeds cannot be dismissed since the QTL may not be segregating in the other breed, i.e. the QTL is breed specific, or its effect may be substantially reduced in a different genetic background. QTLs for skatole are mostly not negatively affecting sex hormones and should be easier to use directly into the current breeding scheme. Generally, the combination of high density SNP genotyping using the 60 K porcine SNP array, GWA and LDLA analysis revealed many genome-wide significant QTLs (25 in total), many of which were confirmed across breeds and tissues where the traits were recorded, and/or method of analysis. Therefore, with the new and more precise information on SNP-effects for androstenone and skatole, we currently suggest a breed specific panel for SNPs affecting androstenone only (not estrogens) and skatole. For further research, more knowledge is needed to determine the relationship between boar- and sow fertility, sex steroids and boar taint compounds to go to the next step of selection using all the genetic markers for androstenone detected in this study. It is a possibility that the effect of the QTLs on the other steroids will reduce boar- and/or sow-fertility, in which case it is a problem for pork production.

## Authors' contributions

EG was coordinating the study, designing the experiment, organizing sample collection, preparation and genotyping experiment, involved in some of the statistical analyses, and drafted the paper. SL was involved in planning the project, provided laboratory facilities and took part in writing the paper. HH and MHSH were performing the preparation of samples, genotyping and quality control of genotypes. MK was responsible for the genotyping facilities, supervising the quality control, and involved in writing the paper. MVS was combining the LDLA and GWA results with the previous gene expression results performed in the same population, involved in discussions and contributed to the paper. THEM was involved in designing the experiment, conducting the statistical analysis and took part in writing the paper. All authors have read and approved the final manuscript.

## Supplementary Material

Additional file 1 **Summary of the genetic maps, SSC1-SSC18, in Landrace and Duroc**. An overview of the lengths of all the autosomal chromosomes (SSCs) in basepairs (bp) and centimorgans (cM), as well as number of SNPs per chromosome in Norwegian Landrace and Duroc. Click here for file
