# Supplementary material for: Large scale genome-wide association and LDLA mapping study identifies QTLs for boar taint and related sex steroids
Source: BMC Genomics. 2011 Jul 13;12:362. doi: 10.1186/1471-2164-12-362 (PMC3149590; doi:10.1186/1471-2164-12-362)
Supplement: Additional file 1 — Summary of the genetic maps, SSC1-SSC18, in Landrace and Duroc. An overview of the lengths of all the autosomal chromosomes (SSCs) in basepairs (bp) and centimorgans (cM), as well as number of SNPs per chromosome in Norwegian Landrace and Duroc. [file 1471-2164-12-362-S1.PDF]

Additional file 1: Summary of the genetic maps, SSC1–SSC18, in Landrace and Duroc.

SSC	No. Bp	Total no. of SNPs	No. of SNPs, Landrace	Length (cM), Landrace	No. of SNPs, Duroc	Length (cM), Duroc
1	289605410	5563	4589	199.8	4287	139.4
2	136831353	2832	2493	132.5	2332	109.7
3	121370645	2409	2057	133.0	1907	97.7
4	135682370	3193	2741	137.2	2747	93.2
5	98654333	1951	1665	115.0	1625	92.4
6	121977168	2665	2385	165.7	2223	133.6
7	133980304	2933	2495	139.9	2540	117.3
8	119003576	2320	2104	135.4	2004	106.1
9	130979392	2747	2494	128.9	2258	102.6
10	64657471	1442	1249	110.3	1236	84.2
11	78997353	1652	1342	82.4	1459	59.0
12	56968097	1342	1252	90.5	1071	64.1
13	145095388	3176	2813	146.8	2516	111.9
14	148457237	3568	3143	134.0	3037	104.4
15	132185762	2390	1909	123.7	1986	96.7
16	76538414	1653	1458	87.7	1379	72.7
17	64042249	1482	1213	80.0	1274	61.2
18	54164418	1162	994	57.8	988	53.8
Total	2109190940	44480	38396	2200.6	36869	1700
